# Hypersensitivity to passive voice hearing in hallucination proneness

**DOI:** 10.3389/fnhum.2022.859731

**Published:** 2022-07-28

**Authors:** Joseph F. Johnson, Michel Belyk, Michael Schwartze, Ana P. Pinheiro, Sonja A. Kotz

**Affiliations:** ^1^Department of Neuropsychology and Psychopharmacology, University of Maastricht, Maastricht, Netherlands; ^2^Department of Psychology, Edge Hill University, Ormskirk, United Kingdom; ^3^Faculdade de Psicologia, Universidade de Lisboa, Lisbon, Portugal; ^4^Department of Neuropsychology, Max Planck Institute for Human Cognitive and Brain Sciences, Leipzig, Germany

**Keywords:** temporal voice area (TVA), voice perception, hallucination proneness, functional magnetic brain imaging (fMRI), neuroimaging, salience account

## Abstract

Voices are a complex and rich acoustic signal processed in an extensive cortical brain network. Specialized regions within this network support voice perception and production and may be differentially affected in pathological voice processing. For example, the experience of hallucinating voices has been linked to hyperactivity in temporal and extra-temporal voice areas, possibly extending into regions associated with vocalization. Predominant self-monitoring hypotheses ascribe a primary role of voice production regions to auditory verbal hallucinations (AVH). Alternative postulations view a generalized perceptual salience bias as causal to AVH. These theories are not mutually exclusive as both ascribe the emergence and phenomenology of AVH to unbalanced top-down and bottom-up signal processing. The focus of the current study was to investigate the neurocognitive mechanisms underlying predisposition brain states for emergent hallucinations, detached from the effects of inner speech. Using the temporal voice area (TVA) localizer task, we explored putative hypersalient responses to passively presented sounds in relation to hallucination proneness (HP). Furthermore, to avoid confounds commonly found in in clinical samples, we employed the Launay-Slade Hallucination Scale (LSHS) for the quantification of HP levels in healthy people across an experiential continuum spanning the general population. We report increased activation in the right posterior superior temporal gyrus (pSTG) during the perception of voice features that positively correlates with increased HP scores. In line with prior results, we propose that this right-lateralized pSTG activation might indicate early hypersensitivity to acoustic features coding speaker identity that extends beyond own voice production to perception in healthy participants prone to experience AVH.

## Introduction

The human voice is a complex signal that carries rich information. This allows the listener not only to identify linguistic messages but also who speaks and how something is said (Belin et al., 2004; Lavan et al., 2019). Some individuals experience auditory verbal hallucinations (AVH), in which they perceive voices in the absence of a corresponding incoming voice signal (Bentall, 1990; Anthony, 2004; Brookwell et al., 2013). Experience of AVH is a key symptom of schizophrenia (Bauer et al., 2011; Larøi et al., 2012; Hugdahl and Sommer, 2018). Yet, it is also reported in multiple other psychiatric, developmental, and neurological disorders (Van Os et al., 2000; Reininghaus et al., 2016; Waters and Fernyhough, 2017; Rollins et al., 2019; Zhuo et al., 2019) and in a minority of otherwise healthy people (Beavan et al., 2011; Linscott and Van Os, 2013; McGrath et al., 2015). Variability in AVH phenomenology exists within and across brain disorders (Stephane et al., 2003; Jones, 2010) and between clinical and non-clinical voice hearers (Daalman et al., 2011; Larøi et al., 2012; Johns et al., 2014; Baumeister et al., 2017). However, hallucinated voices commonly carry information regarding the identity or emotion of a perceived speaker (Stephane et al., 2003; Larøi and Woodward, 2007; Badcock and Chhabra, 2013; McCarthy-Jones et al., 2014), therefore involving a wide range of cortical areas in a voice perception network. Multiple cognitive theories have been proposed delineating the emergence and phenomenology of AVH (Jones, 2010; Ćurčić-Blake et al., 2017; Rollins et al., 2019). One long standing model considers hallucinations as the misattribution of self-generated input to an outside source (Feinberg, 1978). In terms of AVH, signals from voice production cortical regions during inner speech are misperceived as hearing someone else speak (Allen et al., 2007a; Jones and Fernyhough, 2007a,b; Swiney and Sousa, 2014; Gregory, 2016). Recently, competing theories have gained traction, claiming that the initiation of hallucinations does not require motor activity while they are, at their core, misperceived sensations from the environment (e.g., Ford and Mathalon, 2019; Thakkar et al., 2021).

The selection and processing of sensory inputs from the environment relevant to learning, adaptation, or behavioral responses involves multiple regions and distributed networks across the brain. The role of salience attribution within this integrated system provides the necessary trigger to shift processing from a state of rest to active sensation and perception (Menon and Uddin, 2010; Menon, 2011; Palaniyappan and Liddle, 2012; Uddin, 2015). According to this framework, increased auditory cortex activation associated with AVH can be ascribed to a bottom-up hypersensitivity, or salience bias, toward irrelevant sounds. The modulation and over-weighting of top-down predictions may influence this salience bias as well as guide the system to perceive what it expects in meaningless unimodal and multimodal stimuli (Friston, 2005, 2012; Fletcher and Frith, 2009; Deneve and Jardri, 2016; Jardri et al., 2016; Leptourgos et al., 2017). Since voice signals in humans are inherently salient to human listeners, they may be particularly implicated in hypersensitive responses leading to false perceptions. Furthermore, for those who experience AVH, the engagement of brain regions controlling inner speech signals, memory retrieval, and emotion may then guide the phenomenology of the perceived speech in terms of content and speaker-related features (Waters et al., 2012). Abnormal salience processing has been strongly linked to positive symptoms of schizophrenia (Miyata, 2019).

Researching the contribution of these mechanisms to AVH in non-clinical samples may be particularly useful as it avoids potential confounds seen in clinical populations such as medication, age of onset, and duration of symptoms that may affect brain structure and function (Verdoux and van Os, 2002; Kelleher et al., 2010; Kelleher and Cannon, 2011). This perspective is in line with the experiential continuum of psychosis (Johns, 2005; Beavan et al., 2011; Larøi et al., 2012; de Leede-Smith and Barkus, 2013; Johns et al., 2014; Zhuo et al., 2019), whereby functional variability in the mechanisms serving perception across the population account for the spectrum of normal experience, vivid perceptions and imagery, sub-clinical forms of hallucinations, and those seen in full-blown psychosis. The revised Launay-Slade Hallucination Scale (LSHS) is as a measure of perceptual experience and beliefs associated with vivid daydreams, thoughts, imagery, and those related to false perceptions such as visual and auditory hallucinations (Larøi and Van Der Linden, 2005). The LSHS provides a measure of hallucination proneness (HP), where higher scores signify increasing abnormality in perceptual experience and beliefs, including true hallucinations. Although individual items from the LSHS can be used to identify the prevalence of AVH (e.g., Kompus et al., 2015), HP itself is not a measure of risk for psychosis.

Two critical factors have been incorporated into the formulation of our hypotheses. First, differential brain activity may indicate abnormal voice processing as a predisposition for false perceptions, i.e., activation patterns similar to those during hallucinations. Second, the localization of reported changes in brain responses may indicate a specific stage within hierarchical voice processing at which this predisposition manifests. To date, no consensus has been empirically established regarding a trait-based association between hallucinations and brain responses to the voice. For example, when presented with voices, patients who commonly experience hallucinations display decreases (Copolov et al., 2003), increases (Martí-Bonmatí et al., 2007; Parellada et al., 2008; Escartí et al., 2010), or no activation differences in voice selective temporal regions (Woodruff et al., 1997; Simons et al., 2010). Such inconsistency is likely due to methodological heterogeneity (Bohlken et al., 2017). For example, these studies differed in terms of stimulus type, stimulus content, and the inclusion of a non-hallucinating patient control group. Moreover, patients with chronic hallucinations can experience spontaneous AVH during scanning (Jardri et al., 2011; Kühn and Gallinat, 2012; van Lutterveld et al., 2013; Zmigrod et al., 2016), which may even be unintentionally elicited by tasks (e.g., Copolov et al., 2003; Parellada et al., 2008). Although this hallucinatory state elicits brain activity in voice perception regions, simultaneous external voice input during AVH results in a paradoxical net activity decrease (Kompus et al., 2011; Hugdahl and Sommer, 2018).

The localization of changes in functional brain activity within the voice processing network can be particularly informative in determining how HP may arise. Within the upper bank and lateral regions of the temporal lobe, voice signals are processed hierarchically along a pathway composed of multiple functional subsystems or components (Belin et al., 2004; Pernet et al., 2015; Zhang et al., 2021). The engagement of these temporal voice areas (TVA) starts with the evaluation of low-level acoustic features in the posterior superior temporal gyrus (STG), an area specialized in processing spectro-temporal properties of complex sounds (Griffiths and Warren, 2004; Warren J. D. et al., 2005; Warren J. E. et al., 2005). Further processing occurs along hemispherically specialized pathways, with linguistic features predominantly in the left and paralinguistic (i.e., speaker-related information) in the right side of the brain (Belin et al., 2000; Formisano et al., 2008). However, some stimuli such as emotional vocalizations contain both speaker-and speech-relevant information and involve bilateral processing of separate features in the signal (Schirmer and Kotz, 2006). Importantly, AVH often contain marked paralinguistic information about speaker identity or emotion (Larøi and Woodward, 2007; Larøi et al., 2012; McCarthy-Jones et al., 2014). In non-clinical voice hearers, however, the degree of perceived emotional valence is less prominent (Daalman et al., 2012; de Boer et al., 2016). Speaker-related feature processing operates along a multi-stage hierarchy in the right temporal cortex along a posterior to anterior gradient (Nakamura et al., 2001; Belin and Zatorre, 2003; von Kriegstein et al., 2003; von Kriegstein and Giraud, 2004). The TVA localizer is a widely used fMRI task which reliably identifies activation peaks localized in the bilateral anterior, middle, and posterior superior temporal cortex (Pernet et al., 2015). By comparing voice to non-voice activation in response to passively heard sounds, regions of interest (ROI) can be defined for further investigation. Using ROIs produced by this task, we predicted HP-related early sensitivity to low-level voice features to be isolated to the posterior STG ROI. Alternatively, changes to voice processing in the anterior direction of the right STG might indicate an abnormal salience bias for identity or emotion associated with an increasing propensity to hallucinate.

## Methods

### Participants

Twenty-six participants took part in this study, recruited through the SONA system and social media channels at Maastricht University, Netherlands. Participants were provided with informed consent and offered university study credit for compensation. Exclusion criteria included any history of psychotic disorder, neurological impairment, history of drug dependence or abuse, and traumatic brain injury. Participants were screened for MRI safety and reported no metal implants, claustrophobia, or pregnancy. Furthermore, all participants reported no known hearing deficits. Robust statistics using the interquartile range rule for participant age revealed one outlier (Rousseeuw and Hubert, 2011), leading to the exclusion of the dataset from further analysis. Of the resulting 25 individuals (17 female), the average age was 20.92 years (SD 3.95; range 18–32). The Ethical Review Committee of the Faculty of Psychology and Neuroscience at Maastricht University (ERCPN-176_08_02_2017) approved this study.

### Hallucination proneness

The revised LSHS was employed as a self-report measure of HP (Larøi and Van Der Linden, 2005). The questionnaire consists of 16 items targeting tactile, sleep-related, visual, and auditory modalities of psychosis-like experience as well as vivid thoughts and daydreaming. Responses were given using a five-point Likert scale, measuring the extent to which each statement applied to them. The sum of all responses equated to an overall HP measure. Furthermore, to investigate the exclusivity of auditory-only items, subscores of three items were summed to produce a composite score (Larøi et al., 2004; Larøi and Van Der Linden, 2005).

### Voice area fMRI-localizer task

Voice selective cortical brain regions were identified using a standard fMRI-localizer task (Belin et al., 2000). This widely used tool reliably probes activity across three bilateral peaks in the superior temporal gyrus (e.g., Pernet et al., 2015), often designated as anterior, middle, and posterior temporal voice areas (TVA). Furthermore, many studies applying this task have reported extra-temporal voice regions, such as the inferior frontal cortex (IFC). The voice area localizer consists of 20 vocal (V) and 20 non-vocal (NV) trials. Additionally, 20 silence (S) trials are included allowing relaxation of the hemodynamic response to auditory stimuli. The voice condition is composed of human speech (words, syllables, or sentence excerpts) and non-speech voices produced by male and female speakers of different ages (7 babies, 12 adults, 23 children, and 5 elderly). This broad selection of voice stimuli allows for the probing and inclusion of functionally diverse regions of TVA. Conversely, the non-voice condition includes environmental (natural and animal) and man-made (e.g., cars, alarm clocks, instrumental music) sounds. Sound clips are presented at a standard 70 db volume (for a detailed report of the included sounds and recording duration, amplitude, and frequency see Pernet et al., 2015). Trials were presented in a pseudorandom order, each with a duration of eight seconds. With a two second inter-trial interval, the total run time of the task was 10 min.

### FMRI data acquisition

Scanning was conducted using a Siemens 3T Magnetom Prisma Fit equipped with a 32-channel head coil (Siemens Healthcare, Erlangen, Germany), at the Scannexus facilities (Maastricht, Netherlands). Structural whole-brain T1-weighted images were acquired with a single-shot echoplanar imaging (EPI) sequence [field of view (FOV) 256 mm; 192 axial slices; 1 mm slice thickness; 1 mm × 1 mm × 1 mm voxel size; repetition time (TR) of 2250 ms; echo-time (TE) 2.21 ms]. For the functional localizer task, T2-weighted EPI scans were collected (FOV 208 mm; 60 axial slices; 2 mm slice thickness; 2 mm × 2 mm × 2 mm voxel size; TE 30 ms; flip angle = 77°). To reduce scanner noise interference, auditory stimuli were presented *via* S14 MR-compatible earphones, fitted with foam earplugs (Sensimetrics Corporation). Furthermore, to provide relative silence during playback of auditory stimuli, a long inter-acquisition-interval was adopted where time between consecutive acquisition was delayed, resulting in a TR of 10 s. The delayed TR was timed to allow a 2,000 ms acquisition period during peak activation in the auditory cortex (Belin et al., 1999; Hall et al., 1999).

### Data pre-processing and analysis

Pre-processing of the TVA localizer blood-oxygen-level-dependent (BOLD) signal was conducted in SPM12 (Wellcome Department of Cognitive Neurology, London, United Kingdom). A standard pipeline was applied using slice timing correction, realignment and unwarping, segmentation, normalization to standard (MNI) space (Fonov et al., 2009), and 8 mm isotropic Gaussian kernel full width at half maximum (FWHM) smoothing. Analysis followed a two-level procedure in which contrast estimates were first determined as fixed effects at the level of individual participants then modeled as random effects at the level of the sample. Contrast estimates were computed on BOLD data to assess voice sensitivity (V > NV) and sensitivity to environmental sounds (NV > S) for each participant. A first-level fixed-effects GLM analysis for the conjunction analysis [(V > NV) ∩ (V > S)] was computed to localize the temporal voice areas. A second-level random-effects analysis tested for group-level significance and determined the ROIs for parameter extraction. Contrast estimates of V > S and NV > S were then used to contrast voice with non-voice activity, corrected for baseline, in the subsequent hypothesis-driven ROI analysis to investigate the correlation of voice-preferential TVA activity compared to HP. Contrast estimates were extracted from a 5 mm radius of the center coordinates from each region of peak activity produced in the TVA-localizer using the SPM MARSbar toolbox (Brett et al., 2002). Pearson’s correlation analysis using bootstrapping (5000 samples) and bias-corrected confidence intervals was then employed to test for significant relationships between the sensitivity of the voice ROIs and HP measures.

## Results

### Hallucination proneness

For the HP composite score (possible maximum score of 80), the mean self-reported rating was 25.20 (SD 10.47; range 0–42). The HP auditory subscale mean score (possible maximum score 15) was 3.92 (SD 2.74; range 0–11). To test for normality of the distribution of demographics and HP across the sample, Shapiro–Wilk tests were conducted. Both total LSHS (0.948, df = 25, *p* = 0.229) and auditory subscale (0.928, df = 25, *p* = 0.078) were not different from normal. A moderately strong correlation was also found between LSHS auditory subscale and non-auditory item totals (*r* = 0.457, df = 25, *p* = 0.019).

### Voice area localizer

**FIGURE 1:**
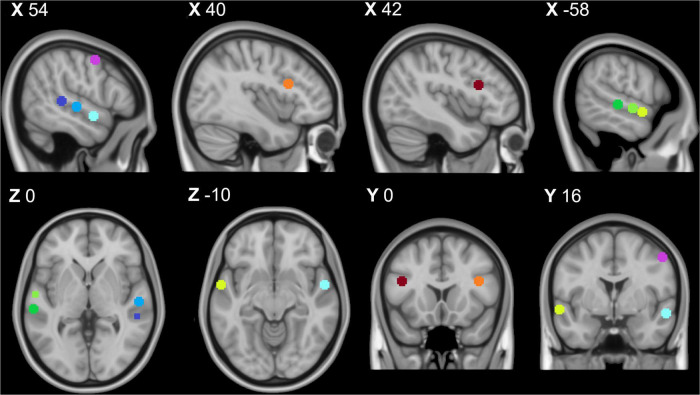
Temporal voice area fMRI localizer task results: Purple = right premotor cortex, dark blue = right posterior temporal gyrus, middle blue = right middle temporal gyrus, light blue = right anterior temporal gyrus, orange = right inferior frontal cortex, dark green = left posterior superior temporal gyrus, middle green = left middle superior temporal gyrus, light green = left anterior superior temporal gyrus, and red = left inferior temporal cortex. All coordinates listed in MNI space (x,y,z). This image was created using the FSL toolbox fsleyes (McCarthy, 2022).

The fMRI localizer task produced 5 clusters covering bilateral lateral temporal cortices, bilateral inferior frontal gyri, and the right precentral gyrus (preCG) (Table 1 and Figure 1). Within each bilateral temporal cortex “voice patch,” peak activity localizations were distinguished in three distinct regions: posterior (pSTG), middle (mSTG), and anterior STG (aSTG). These regions correspond to the expected divisions of the TVA localizer (Pernet et al., 2015).

**TABLE 1:** Results from temporal voice area fMRI localizer task.

Cluster #	Hem.	Label	BA	x	y	z	Cluster-Level p-FDR	Peak-Level p-FDR	Size (voxels)
1	L	mSTG	22	−58	−10	−4	1.6782E-17	1.4637E-09	4145
		pSTG	22	−60	−26	0		1.4637E-09	
		aSTG	22	−58	0	−8		1.3575E-08	
2	R	mSTG	22	56	−18	−2	2.0689E-17	1.4637E-09	4010
		aSTG	22	56	0	−12		1.6043E-08	
		pSTG	22	54	−34	4		1.6043E-08	
3	R	pMC	6	52	2	48	0.0049	4.1457E-05	285
4	L	IFC	44	−42	16	22	0.0383	0.0018	142
5	R	IFC	44	40	16	22	0.0227	0.0302	180

Hem, hemisphere; (a/m/p) STG, (anterior/middle/posterior) superior temporal gyrus; pMC, premotor cortex; IFC, inferior frontal cortex; BA, Brodmann’s Area; p-FDR, false discovery rate corrected *p*-value (threshold = 0.05). All coordinates listed in MNI space (x, y, z).

### FMRI correlation

**FIGURE 2:**
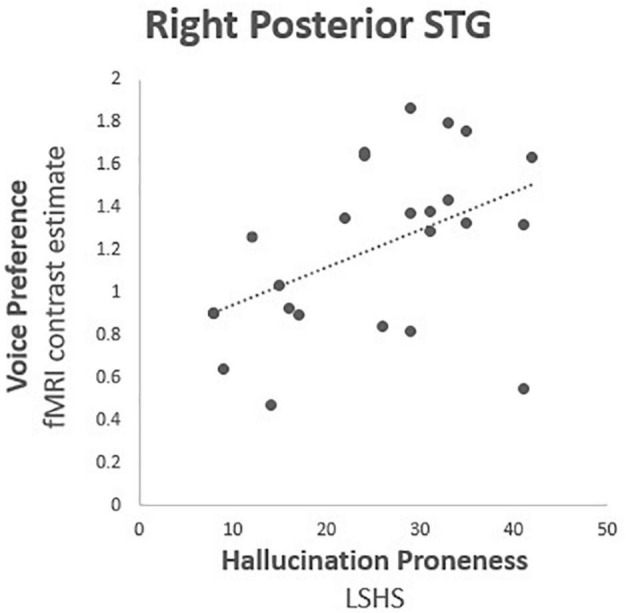
Hallucination proneness fMRI correlation analysis results: Right posterior superior temporal gyrus (BA 22; MNI 54, −34, 4), Voice preference = contrast estimate [(Voice > Silence) > (Non-voice > Silence)], LSHS = Launay Slade Hallucination Proneness scale. Correlation coefficient *r* = 0.470, *df* = 25, *p* = 0.020.

Correlational tests were performed between contrast estimates representing voice preference [(V > S) > (NV > S)] observed in each TVA-ROI with both the composite HP score and the auditory subscore of the LSHS. All thresholds for significance were Bonferroni-adjusted for multiple comparisons using (*p* < 0.025). Only the right pSTG reached statistical significance (*r* = 0.470, df = 25, *p* = 0.020) (Table 2 and Figure 2). *Post hoc* correlation analyses were run to assess the relative contributions of both voice (V > S) and non-voice (NV > S) contrasts to correlational analyses (see detailed results in Supplementary Material). We conducted these analyses in order to rule out a general hypersensitivity of temporal cortex activity non-specific to the conditions of interest probed by the conjunction analysis. No significant correlations with HP were found in any ROI for voice (V > S), however, a significant negative correlation was reported in the right IFC for non-voice (V > S) sensitive activity (*r* = −0.614, df = 25, *p* = 0.001).

**TABLE 2:** Voice preference response [(Voice > Silence) > (Non-voice > Silence)] correlation with hallucination proneness results.

		ROI			LSHS		LSHS-Auditory	
Hem.	Label	μ	SD	CI (95%)	*r*	*p*	*r*	*p*
L	aSTG	1.189	0.479	0.203–0.434	0.120	0.576	0.178	0.406
	mSTG	1.505	0.586	0.997–1.380	−0.237	0.267	−0.024	0.915
	pSTG	1.511	0.560	1.271–1.740	−0.058	0.791	0.055	0.797
R	aSTG	1.019	0.452	0.838–1.200	0.266	0.208	0.165	0.440
	mSTG	1.295	0.515	1.089–1.501	−0.177	0.408	−0.033	0.882
	pSTG	1.213	0.406	1.051–1.375	0.470	* *0.020*	0.276	0.192
R	pMC	0.625	0.447	0.446–0.804	0.087	0.685	−0.103	0.635
L	IFC	0.319	0.288	0.204–0.434	−0.048	0.827	−0.025	0.911
R	IFC	0.293	0.323	0.164–0.422	0.231	0.277	0.134	0.534

ROI, region of interest; (a/m/p) STG, (anterior/middle/posterior) superior temporal gyrus; pMC, premotor cortex; IFC, inferior frontal cortex; μ, mean activation from contrast; SD, standard deviation; LSHS, Launay-Slade Hallucination Proneness scale; LSHS-Auditory, subset of 3 auditory items, r = correlation coefficient, Bonferroni-corrected significance level (*p < 0.025).

## Discussion

The current study investigated whether a measure of abnormal perceptual experience (HP) in a non-clinical sample is associated with variability in the functional brain responses of the temporal cortex regions serving detecting and processing of voice signals. Considering the well-established roles of specific voice sensitive regions of the cerebral cortex, we aimed to determine if this putative relationship would be limited to specific subprocesses in hierarchical voice perception. As hypothesized, activity for voice versus non-voice processing correlated positively with HP only in the pSTG, a region associated with the early processing of low-level acoustic features in complex auditory signals (i.e., Griffiths and Warren, 2004; Warren J. D. et al., 2005; Warren J. E. et al., 2005). Furthermore, this finding was restricted to the right hemisphere and therefore is likely linked to the processing of paralinguistic voice information (Belin et al., 2000; Formisano et al., 2008). Additionally, *post hoc* analysis revealed a negative correlation with HP in the right IFC for non-voice versus silence. Together, these findings may confirm that as the propensity to hallucinate increases, right posterior temporal lobe voice hypersensitivity increases and is accompanied by a decreased prefrontal response to non-vocal environmental sounds.

### Hallucination proneness and hypersensitivity

Multiple neurocognitive mechanisms underlying hallucinations have been proposed. Most commonly, these theories have focused on describing the emergence and phenomenology of pathological voice hearing in patients with psychotic disorders such as schizophrenia (Allen et al., 2008; Hugdahl, 2015; Ćurčić-Blake et al., 2017). The most influential models describe atypical increases in brain activity in cortical voice regions. The current investigation was approached from the perspective of perceptual salience models claiming a central role of hypersensitivity to irrelevant sensory stimuli in auditory regions (Menon and Uddin, 2010; Menon, 2011; Palaniyappan and Liddle, 2012; Uddin, 2015). Conversely, prominent self-monitoring models of hallucinatory experience describe increased activity as the result of insufficient suppression of sensory cortices during inner speech (Frith and Done, 1988; Weiss and Heckers, 1999; Tracy and Shergill, 2006; Allen et al., 2007a,^2008^; Jones and Fernyhough, 2007b). According to this theory, the activation of speech production regions is required for the emergence of AVH. However, the current results demonstrate that variability in voice processing cortical regions in relation to HP exists without motor activity.

It is possible that theories proposing divergent involvement of speech production and perception mechanisms in AVH may be not mutually exclusive. Experiences of people who hallucinate are diverse. As theories of HP become more specific and concrete, they may become less well aligned with the phenomenology of the hallucinator. Therefore, hallucinatory experience might be best characterized by multiple subtypes, to which specific theories might apply better than others (Jones, 2010). For example, models describing the phenomenology of voice hearing ascribe the top-down contribution of intrusive memories and thoughts to the quality of false perception experiences (Hugdahl, 2015; Upthegrove et al., 2016; Bohlken et al., 2017; Ćurčić-Blake et al., 2017). A core abnormality in brain function central to the emergence of false perceptions likely rests in the interactive process of top-down predictions and bottom-up sensory input (Allen et al., 2008; Hugdahl, 2009, 2015; Kowalski et al., 2021). Regarding perceptual salience, bottom-up hypersensitivity to sensory input is congruent with established computation neuroscience accounts of predictive coding in false perceptions (Sterzer et al., 2018). Here, weighted top-down predictions and bottom-up explanations of sensation interact along a hierarchical network, constantly updating *via* Bayesian inference to form the most reliable percept (Friston, 2005, 2012; Fletcher and Frith, 2009; Feldman and Friston, 2010; Hohwy, 2017). When internal prediction signals are weighted too strongly, one “senses what they expect.” Moreover, when the top-down input is too strong, the threshold for active perception may be reached under minimal sensory input. However, the self-monitoring theory posits a delayed or absent prediction signal resulting in increased activation of sensory cortical regions and is therefore in apparent conflict with the former account (Corlett et al., 2019; Leptourgos and Corlett, 2020). These expectations could operate on separate time scales, at different levels of the information processing hierarchy, or simply serve two different functions in hallucinations (Thakkar et al., 2021).

The role of perceptual salience in a multistage process leading to false perceptions has gathered substantial support in functional neuroimaging. Namely, research into large-scale functional brain networks has provided a resting-state hypothesis, outlining brain states serving as a predisposition for hallucinations, including voice hearing (Northoff and Qin, 2011; Northoff, 2014). While at rest, activation of the salience network, under conditions of irrelevant stimuli, may interrupt the Default Mode Network (DMN) and engage active sensory processing (Alderson-Day et al., 2015, 2016; Schmidt et al., 2015). The salience network therefore operates as a switch between the DMN and central executive network and how attention is directed toward incoming sensations, constituting a triple network model (TMN) subserving the advent of hallucinatory experience (Menon, 2011). Although we did not acquire behavioral data from the participants with ratings of perceived salience while listening to stimuli during scanning, we suggest that the change in brain activity that we observed in the right pSTG is indicative of the TMN in response to voice stimuli.

### Hierarchical voice network processing

Voices are processed along a series of bilateral voice patches in the posterior, middle, and anterior STG. These temporal voice areas are reliably identified by a standardized TVA localizer task (Pernet et al., 2015). Participants with greater HP displayed increased right pSTG activation in response to vocal stimuli. Activity in this region may reflect sensitivity to low-level acoustic features during early stages of voice processing (Griffiths and Warren, 2004; Warren J. D. et al., 2005; Warren J. E. et al., 2005). Furthermore, the pSTG is not specialized for voice processing *per se*, and likely plays a broader role in extracting spectro-temporal acoustic features from complex sounds, of which voices are an example. However, activation in these regions preferentially responds to salient stimuli, such as voices, over and above other similarly complex environmental sounds (Pernet et al., 2007).

In terms of the salience hypothesis for hallucinatory experience, the assignment of salience to irrelevant, neutral, events must be considered in terms of the paralinguistic factors which may be involved. Indeed, the phenomenology of AVH is often marked by prominent paralinguistic features in the identity and emotional valence of the hallucinated speaker (Stephane et al., 2003; Larøi and Woodward, 2007; Badcock and Chhabra, 2013; McCarthy-Jones et al., 2014). Individuals who experience hallucinations often express difficulty in discerning the identity of veridical voices. For example, in schizophrenia patients who experience hallucinations, there is a bias to externalize voices to another person (Johns et al., 2001; Allen et al., 2007b; Mechelli et al., 2007; Pinheiro et al., 2016, 2017). Likewise, severity of AVH in patients is increasingly altered by emotional processing (Rossell and Boundy, 2005; Shea et al., 2007; Alba-Ferrara et al., 2013; Tseng et al., 2013). The role of salience may be influential in perceptions of speaker identity, as misattributions are more prevalent for emotional stimuli (Ditman and Kuperberg, 2005; Costafreda et al., 2008; Pinheiro et al., 2016, 2017). However, the effects of emotional valence in perceiving voice identity for people prone to false perceptions of voices has not shown clear consensus (i.e., Brookwell et al., 2013). Comparisons of AVH severity in patients with schizophrenia with judgments of speaker identity have indicated an increasing proneness to externalize voices with negative content (Allen et al., 2004; Pinheiro et al., 2016). In non-clinical groups, the involvement of salient emotional features in voices is less clear. For example, higher levels of HP in the general population are not associated with atypical evaluation of emotional valence in words or vocalizations (Pinheiro et al., 2019). However, it has been indicated that non-clinical individuals prone to voice hearing require stronger emotional information to consider a stimulus as emotional (Amorim et al., 2021) or may allocate similar attention to voices irrespective of their emotional salience (Castiajo and Pinheiro, 2021). Future research is required into how variability in perceived salience of speaker-related features may affect processing in the hierarchical voice network and, in particular, how posterior STG activity related to HP may be influenced.

In addition to the TVA findings, the localizer task often provides a subset of extra-temporal regions indicating an extended voice processing network (Pernet et al., 2015). In our sample, extra-temporal peak activations were ascribed to bilateral inferior frontal and right hemisphere premotor cortex. Prefrontal involvement of the left IFC is commonly found in voice perception, with different subregions serving various functions. For example, the pars orbitalis is involved in processing semantic and emotional information (Belyk et al., 2017). Here, the left IFC peak was found in Broca’s area, which has been theorized to represent mirror neuron activity which may be useful in guiding conversational turn-taking (Rizzolatti and Craighero, 2004; Grafton and Hamilton, 2007; Kilner et al., 2007). Likewise, precentral motor regions are involved in the perception and production of speech (Wilson et al., 2004; Pulvermüller et al., 2006; Cheung et al., 2016). This could explain speech production region activity sometimes reported during AVH (Jardri et al., 2011; Kühn and Gallinat, 2012; Zmigrod et al., 2016). However, self-monitoring theories take this as evidence for top-down inner speech signals guiding the perceived hallucinatory voice. Notably, transcranial direct-current stimulation targeting a fronto-parietal sensorimotor network is an effective treatment for the alleviation of AVH in patients with schizophrenia (Yang et al., 2019). In our *post hoc* analysis, the right IFC ROI shows an intriguing negative correlation to HP, however, only for non-voice sounds. The right IFC may serve a role in salience processing, for example in recognizing salient cues in voice signals (Johnstone et al., 2006; Bestelmeyer et al., 2012; Charest et al., 2013; Johns et al., 2015; Johnson et al., 2021). Additionally, this area shares a high functional integration with temporal regions serving voice perception and may assist successful voice recognition (Aglieri et al., 2018). Although this finding is difficult to interpret on its own, it may indicate a decrease in salience attribution for environmental sounds during a voice perception task. This may indicate not only an HP-related salience bias affecting the sensitivity of cortical responses to voice sounds, but also a general bias away from non-voice sounds between hypersalient responses to intermittent voice stimuli.

### Limitations and recommendations

We identify a number of limitations within the current study and provide suggestions for future research. First, although the use of the established TVA localizer task facilitated the testing of our hypotheses regarding an early hypersensitivity to voice sounds, it did not preclude further investigation into how more complex stages of the voice processing hierarchy may relate to HP. Specifically, BOLD responses from this task are averaged across the trials containing different types of voice stimuli. This implies that signals extracted from ROIs serving different functional roles in voice processing, e.g., emotion or identity, do not represent the processing of specific features, but rather constitute a generalized voice detection signal. Second, in this study, behavioral measures of perceived stimulus salience were not collected. Therefore, interpretations of a salience bias attributed to increased functional brain responses cannot be directly linked to the subjective perception of the participants. Third, participants in the current study were sampled from a relatively homogenous sample of university students, similar in age, ethnicity, and cultural backgrounds. Due to the uneven distribution of environmental risk factors for psychotic symptoms throughout the population (Johns and van Os, 2001; DeRosse and Karlsgodt, 2015; Baumeister et al., 2017), our sample may unintentionally capture a set of protective factors. To address these limitations in future studies, we suggest a two-step procedure using a novel task that systematically varies paralinguistic voice features. This may allow investigations into how hierarchical processing downstream of initial HP-related hypersensitivity may influence responses to the perceived emotion or identity of the speaker. Furthermore, behavioral appraisals of perceived salience may be included to compare fMRI response patterns and HP scores. Finally, subsequent research may benefit from an increased sample size and diversity, including a structured collection of additional demographic data and associated environmental risk factors as possible covariates for HP-related brain changes.

## Conclusion

We observed that HP is positively correlated with increased activation in the right pSTG in response to passively heard voices. This suggests a hypersensitivity associated with a propensity to hallucinate in a region of the brain which extracts low-level acoustic features from complex auditory signals. The right pSTG comprises the early processing of voice signals along the paralinguistic information pathway of the cortical voice processing network. We propose that this increases activity in response to voices represents a perceptual salience bias as a precursor for the emergence of hallucinations. This interpretation is in line with functional network models that posit abnormal engagement of a salience network during irrelevant stimulus exposure as the underlying neurocognitive mechanism of false perceptions. Furthermore, the current findings conflict with self-monitoring accounts of inner speech models that propose a critical role of voice production regions in the inception of AVH. We have demonstrated that HP is associated with right pSTG activation driven by external auditory signals. Although we do not reject self-monitoring accounts, we suggest that a state of cortical hypersensitivity to irrelevant sensory input may be the first step in the emergence of a hallucinatory experience, possibly followed by the influence of top-down signals such as inner speech, memory, and thought that together contribute to the phenomenology of AVH.

## Data availability statement

The raw data supporting the conclusions of this article will be made available by the authors, without undue reservation.

## Ethics statement

The studies involving human participants were reviewed and approved by the Ethical Review Committee of the Faculty of Psychology and Neuroscience at Maastricht University. The patients/participants provided their written informed consent to participate in this study.

## Author contributions

JJ conceptualized and carried out experiment, performed the analyses, and wrote manuscript with input from all authors. MB verified the analytical methods. SK, AP, MS, and MB conceptualized and interpreted the results. SK and AP provided the original idea for project and secured funding. All authors contributed to the article and approved the submitted version.
