# Supplementary material for: Hypersensitivity to passive voice hearing in hallucination proneness
Source: Front Hum Neurosci. 2022 Jul 28;16:859731. doi: 10.3389/fnhum.2022.859731 (PMC9366353; doi:10.3389/fnhum.2022.859731)
Supplement: Supplementary file 1 [file Table_1.DOCX]

Supplementary Material

**Table 1**. Voice selective response (Voice>Silence) correlation with hallucination proneness results: ROI = region of interest, (a/m/p) STG = (anterior /middle /posterior) superior temporal gyrus, pMC = premotor cortex, IFC = inferior frontal cortex, μ = mean activation from contrast, Min = minimum score; Max = maximum score, SD = standard deviation, LSHS = Launay-Slade Hallucination Proneness scale

| **Voice Sensitive Response Correlation** | | | | | | | |
| --- | --- | --- | --- | --- | --- | --- | --- |
| ***ROI*** | | | | | | ***LSHS*** | |
| ***Hem.*** | ***Label*** | ***μ*** | ***Min*** | ***Max*** | ***SD*** | ***r*** | ***p*** |
| L | aSTG | 1.189 | 0.398 | 2.288 | 0.479 | -0.255 | 0.229 |
|  | mSTG | 1.505 | 0.470 | 2.650 | 0.586 | -0.210 | 0.327 |
|  | pSTG | 1.511 | 0.250 | 2.600 | 0.560 | -0.096 | 0.655 |
| R | aSTG | 1.019 | 0.180 | 1.720 | 0.452 | 0.026 | 0.903 |
|  | mSTG | 1.295 | 0.440 | 2.500 | 0.515 | -0.128 | 0.551 |
|  | pSTG | 1.213 | 0.471 | 1.867 | 0.406 | 0.095 | *0.660* |
| R | pMC | 0.625 | -0.290 | 1.510 | 0.447 | -0.174 | 0.419 |
| L | IFC | 0.319 | -0.273 | 1.009 | 0.288 | -0.190 | 0.376 |
| R | IFC | 0.293 | -0.440 | 0.920 | 0.323 | -0.385 | 0.064 |

**Table 2**. Non-voice selective response (Non-voice>Silence) correlation with hallucination proneness results: ROI = region of interest, (a/m/p) STG = (anterior /middle /posterior) superior temporal gyrus, pMC = premotor cortex, IFC = inferior frontal cortex, μ = mean activation from contrast, Min = minimum score; Max = maximum score, SD = standard deviation, LSHS = Launay-Slade Hallucination Proneness scale

| **Non-voice Sensitive Response Correlation** | | | | | | | |
| --- | --- | --- | --- | --- | --- | --- | --- |
| ***ROI*** | | | | | | ***LSHS*** | |
| ***Hem.*** | ***Label*** | ***μ*** | ***Min*** | ***Max*** | ***SD*** | ***r*** | ***p*** |
| L | aSTG | 1.189 | 0.398 | 2.288 | 0.479 | -0.326 | 0.120 |
|  | mSTG | 1.505 | 0.470 | 2.650 | 0.586 | -0.086 | 0.689 |
|  | pSTG | 1.511 | 0.250 | 2.600 | 0.560 | -0.088 | 0.683 |
| R | aSTG | 1.019 | 0.180 | 1.720 | 0.452 | -0.145 | 0.499 |
|  | mSTG | 1.295 | 0.440 | 2.500 | 0.515 | -0.050 | 0.817 |
|  | pSTG | 1.213 | 0.471 | 1.867 | 0.406 | -0.106 | *0.622* |
| R | pMC | 0.625 | -0.290 | 1.510 | 0.447 | -0.329 | 0.118 |
| L | IFC | 0.319 | -0.273 | 1.009 | 0.288 | -0.208 | 0.329 |
| R | IFC | 0.293 | -0.440 | 0.920 | 0.323 | -0.614 | 0.001 |

**
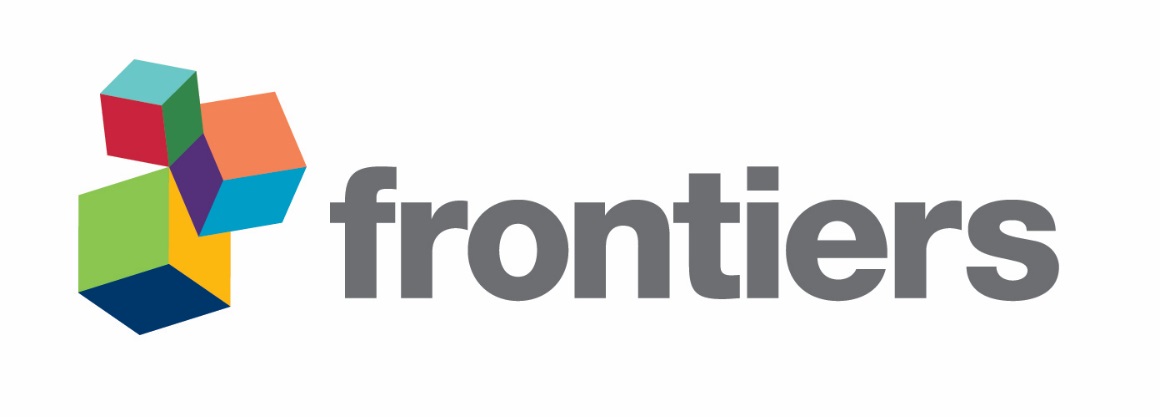
**
